# Feasibility of a new free mobility procedure to evaluate the function of the autonomic nervous system in patients with syncope

**DOI:** 10.1038/s41598-020-70701-y

**Published:** 2020-08-19

**Authors:** Juan Nader-Kawachi, Paulo C. Manrique-Mirón, Yaima C. Pino-Peña, María L. Andrade-Magdaleno, Jesús López-Estrada

**Affiliations:** 1 Cerebrovascular Clinic, Hospital Médica Sur, Puente de Piedra 150-PB, T2, 14050 Mexico City, Mexico; 2 Cátedra-CONACyT, Consejo Nacional de Ciencia y Tecnología (CONACyT), Mexico City, México; 3 Mathematical Applications Laboratory, Institute of Mathematics, UNAM, Cuernavaca, Morelos Mexico; 4 Sciences School, National University of México, Mexico City, Mexico

**Keywords:** Biophysical methods, Cardiovascular diseases

## Abstract

To propose a new test to evaluate the autonomic nervous system in patients with syncope: Multimodal Monitoring for Diagnosis of Dysautonomia (MMDD). We included 21 patients with syncope (16 female, 6 male, mean age 43.5 years) and 21 with no-syncope subjects (15 female, 7 male, mean age 45.1 years) to perform a test of nine 2-min stages: four while resting and four during active testing of autonomic response. Transcranial-Doppler, electrocardiogram, and photoplethysmography blood pressure pulse-to-pulse monitoring, allow registering six variables from the Middle Cerebral Artery and four from the Cardiovascular System. We analyze each variable's mean differences in each stage and its change when they pass from one stage to another with the T and Z tests. To understand the significance of the change, we use a logistic regression model for a certain subgroup of variables. Since we have a small dataset, we use the bootstrap technique to infer the general behavior that characterizes a syncope. Our data confirm differences between syncope and non-syncope patients during MMDD stress stages 2, 4, 6 and 8. Bootstrap and multivariate logistic regression allow us to identify which sets of variables in each of these stages of the MMDD are sufficiently sensitive to recognizing syncope. MMDD protocol can recognize a syncope patient with some confidence by detecting subtle changes in the autonomic nervous system. This protocol encourages us to continue to study the effectiveness of MMDD protocol allowing a new approach to future research.

## Introduction

*Dysautonomia*, defined as changes in the functioning of the autonomic nervous system that negatively affect health status, has manifestations ranging from symptoms located in some part of the body or transient and occasional episodes of neurally mediated hypotension to progressive neurodegenerative diseases^1^. Syncope, the most common event to translate dysautonomia, is defined as a state of transient loss of consciousness of rapid onset, short duration, and spontaneous recovery due to a transitory state of cerebral hypoperfusion that leaves no neurological sequelae^2,3^.

In the Framingham study, at least 3% of men and 3.5% of women between 30 and 62 years old, had an episode of syncope through 26 years follow-up^4^. In 50% of the cases, the clinical history allows us to identify the causes of syncope^5^; however, in the rest, diagnosis of the primary and less understood forms of syncope are elusive^6^.

The phenomenon of syncope occurs because of a state of transient global cerebral hypoperfusion that, independently of the mechanism that initiates it, responds to a decrease in the activity of vasoconstrictor neurons and an increase in the parasympathetic activity of cardiomotor neurons^7^. Due to its complexity, we find several proposals to evaluate the Autonomic Nervous System (ANS) in patients with syncope, most of which study the reactivity of the heart and blood pressure in response to maneuvers such as Valsalva, deep breathing, isometric hand contraction with a dynamometer, orthostatism test, thermal test and, more recently, the analysis of heart rate variability^8,9^. In 1985, Ewing proposed a battery using some of these elements for the study of diabetic autonomic neuropathy, which is in use today^10^.

The Tilt Table Test (TT) is in use since 1986 to understand and classify patients with sincope^11^. To consider positive this test of a passive movement against gravity, the syncope or, at least, the dysautonomic symptoms that precede it, must be triggered, using blood pressure and heart rate as explanatory variables^8,12^. The TT considered a non-invasive procedure associated with few complications. However, historically, new pharmacological tests with nitroglycerin and isoproterenol have been added^13–15^, as well as other maneuvers, such as carotid massage and compression of the eyes^16^, to enhance the test gives TT a certain degree of intervention, most of them are out of use. Even if passive movement in this test eliminates the abrupt changes in heart rate and blood pressure of the regular bodily activity, this type of mobility rarely happens in everyday life.

Transcranial Doppler (TCD) allows studying changes of cerebral blood flow of the Middle Cerebral Artery (MCA) with passive position changes^17,18^, and active orthostatism^19^. The technique of the measurement of the Pulse of Transit Time using the electrocardiogram and photo-plethysmography by infrared light allows recording the arterial blood pressure in each heartbeat^20,21^. TCD and other different ways of recording beat-to-beat pressure have added to the TT protocol^12,22^.

Even considering these statements, the validity and usefulness of TT is debatable as, until now, the induction of syncope or premonitory symptoms of syncope are mandatory to determine when the test is positive. Also, indications for a TT test are limited and appear to have questionable sensitivity and specificity^23^. That is why we do not consider the TT as a gold standard.

According to our hypothesis, subtle body movement causes changes in the autonomic nervous system that can be recorded with multimodal monitoring. The data obtained can be analyzed and amplified using, from simple statistics to complex mathematical models. Our team created a free mobility protocol called Multimodal Monitoring for Diagnosis of Dysautonomia (MMDD)^24^. In this protocol, we recorded ten physiological variables during nine stages in different body positions. We aim to demonstrate that our results, obtained by simple statistical analysis (logistic regression and bootstrap technique), can generate numeric values in people that differentiate who may have syncope.

## Subjects and methods

### Sample selection

We obtained data from 22 consecutively treated patients in the neurology outpatient clinic of Médica Sur Hospital, Mexico City, between July 2015 and January 2016, whose motive for consultation and diagnosis was a first-ever syncope^2,3^. All patients had a cardiological review to discard any cardiological condition related to syncope. All patients had a normal electroencephalogram, glucose tolerance test, and imaging studies (Computerized axial tomography or Magnetic resonance imaging) to rule out any other neurological conditions related to loss of consciousness. We also included 22 control subjects with no syncope or any other state of transitory loss of consciousness matched for age and gender as controls to achieve two homogeneous groups. For both groups, we excluded patients with an apparent potential cause of syncope or a concomitant condition that could affect the Autonomous Nervous System (ANS). We did not include two patients for the final analysis, one in the syncope group and his equivalent from the control group, because of incomplete data.

We performed all the procedures before noon in a closed room with a temperature between 23 ºC and 24 °C. In preparation for the process, we requested the patients to have a light breakfast and, to stop medications that could interfere with the results 48 h before their visit.

### Equipment

We used a Sonara TCD monitoring equipment with two 3 MHz Doppler ultrasonic probes and the respective tailored headband (Viasys Health-Care, Pennsylvania USA) and a Somno-Touch wrist monitor (Somnomedics, Randersacker, Germany).

### Process

1. We start the procedure placing four electrodes on the patient's chest to obtain the electrocardiogram recordings and one infrared digital sensor for the photo-plethysmographic recording of pulse curve, oxemia, and beat to beat blood pressure (mm Hg). At the same time, two 3 Hz Doppler ultrasonic probes fixed with a headband to record, using a transsphenoidal approach, the blood flow velocity (cm/s) of the Middle Cerebral Artery (MCA) on both sides.
2. Once the sensors and devices are confirmed to be working correctly, the test begins.
3. MMDD includes nine positions (Fig. 1). Stages 1, 3, 5, 7, and 9 are while resting (laying down), to reach a baseline between the test movements. Test stages 2, 4, 6, and 8 evaluate the response of different positions to activate the sympathetic nervous system. During stage 2 (sitting with the legs in horizontal position), the isolated effect of changing the trunk position is studied. During stage 4 (sitting with the feet hanging), venous pooling increases to decrease the preload. During stage 6 (standing), the action of the muscles of the lower limbs and the abdomen to decrease the effect of venous pooling and to test orthostatism. Finally, during stage 8, the subject performs physical activity: 15 genuflections or 15 ascents of a step; this directly stimulates adrenergic response.

**Figure 1:**
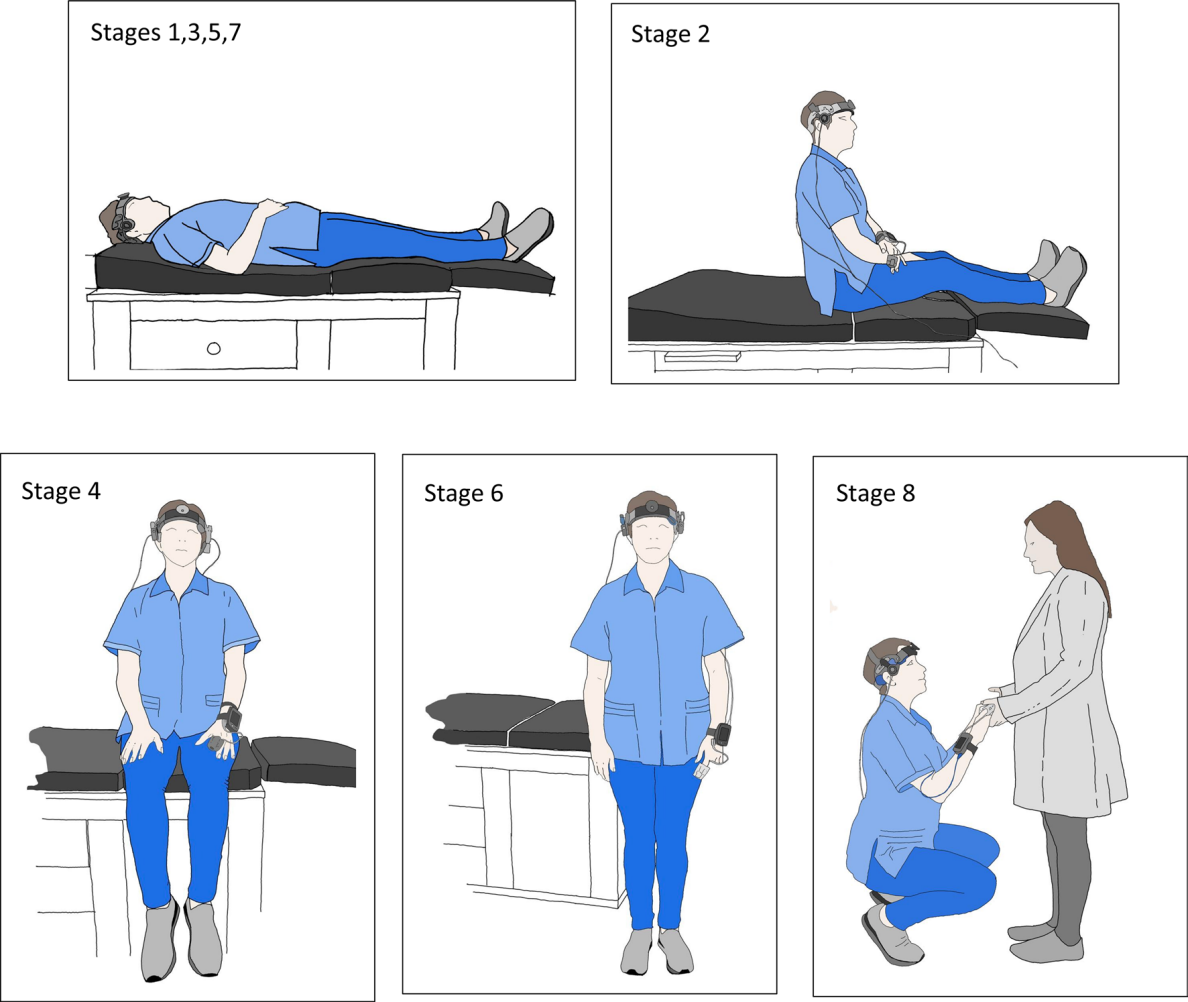
MMDD procedure: Resting stage: Stages 1, 3, 5, 7 (laying down). Testing stages Stage 2; sitting with the legs in the horizontal position, Stage 4; sitting with the feet hanging. Stage 6; Standing up. Stage 8; physical activity (15 genuflections or 15 ascents of a step).
4. With the return to a horizontal position, we measure the capacity of the variables to recover to the basal state. We decided to set a 2-min time for each stage as we consider this a reasonable time to detect the ANS's adaptive changes.

Through mathematical modeling, we compare and analyze the subtle behavioral differences in the variables between patients and controls to sustain that the MMDD is significantly plausible from the statistical point of view.

### Monitoring

With these resources, it was possible to obtain a continuous record of the following variables of each heartbeat: Cerebral Blood Flow (CBF) variables: Systolic velocity of the MCA (Peak-vel.), Mean velocity of the MCA (Mean-vel.), End Diastolic Velocity of the MCA (EDV), Pulsatility Index (PI), Resistance Index (RI), and Systolic/Diastolic MCA velocity index (SD). Cardiovascular variables: Systolic Blood Pressure (SBP), Diastolic Blood Pressure (DBP), and these data allow us to calculate Mean Blood Pressure (MBP) during the procedure. With the electrocardiogram record, we obtained the R-R time used to calculate the Heart Rate (HR) and to rule out cardiac rhythm alterations during the procedure.

We used a specially created software to insert the obtained data in our database automatically and sorted in the different stages using XLSTAT version 15.28 and the programming language R version 3.5.3 (2019-03-11) for statistical proposes.

### *Statistical analysis* (Fig. 2)

**Figure 2:**
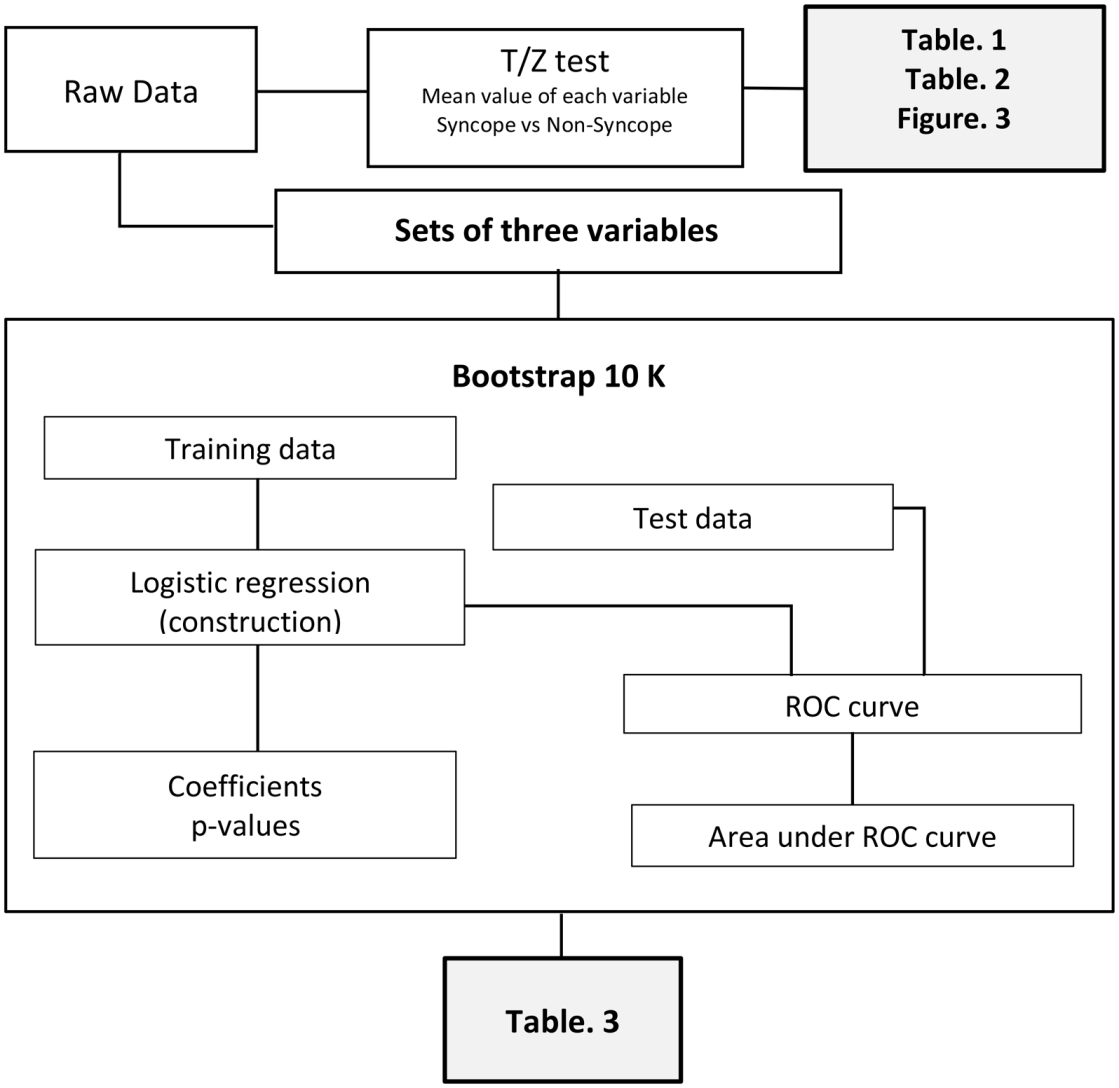
Statistical procedures.

Under the hypothesis that the variables follow a normal distribution (Kolmogorov–Smirnov test), we used the T and Z tests to compare the demographic characteristics between the two groups and be able to consider the samples as comparable. To analyze the variables, we initially took the averages of the physiological variables for subjects with syncope and non-syncope of each two-minute stage. We ran a hypothesis test to determine if there is statistical evidence that the groups are different. Subsequently, the difference between the value of one stage j and the next stage j + 1 is taken, where j = 1, 3, 5, 7, and 9 are at rest and j = 2, 4, 6, and 8 are tested. The resulting value had the suffix "D" (D values) added. We calculated the average D value of the contiguous stages of syncope and non-syncope in each two-minute periods and carried out a hypothesis test to determine the presence of statistical evidence of differences. Finally, with these D values, we constructed a logistic regression model to understand the contribution of the variables with position changes to the description of syncope.

We included data of 42 people out of 44 with the 10 variables with the suffix D in the 9 position changes.

Since the dataset is small, we used the bootstrap method with replacement in order to understand the underlying randomness in the sample. We divided the data into two subsets: training data (to build a logistic regression, a random sample with replacement with the same size as the original data), and test data (to measure the accuracy of the logistic regression, the data which are not selected in the random sample)^25^.

We selected subsets of variables for constructing the logistic regression under the following criteria:

1. For the moment of the cardiac cycle: during the systole (SBP.D, Peak-vel.D, PI.D), during diastole (DBP.D, EDV.D, RI.D) and during intermediate moments (MBP.D, Mean-vel.D, SD.D).
2. By its functional relationship: brain (Peak-vel.D, Mean-vel.D, EDV.D), (RI.D, PI.D, SD.D), systemic (SBP.D, DBP.D), and (SBP.D, HR.D, RI.D).

The equation of the logistic regression:

$${\text{P}}\left( {{\text{y}} = {1}|{\text{x}}_{{1}} ,{\text{ x}}_{{2}} ,{\text{ x}}_{{3}} } \right) \, = { 1}/ \, ({1} + {\exp}( - \, (\beta_{0} + \beta_{{1}} {\text{ x}}_{{1}} + \beta_{{2}} {\text{x}}_{{2}} + \beta_{{3}} {\text{x}}_{{3}} )),$$

where the variables x_1,_ x_2_, and x_3 _correspond to some of the mentioned combinations. The response variable y is binary, where y = 1 is *syncope* and y = 0 is no *syncope*.

We recorded the values of the coefficients β_0_, β_1_, β_2_, and β_3_, the p-value corresponding to the hypothesis that a coefficient is zero. We defined the capacity of the logistic regression to classify (distinguish syncope from no syncope) by the area under the ROC curve.

### Ethical standards

MMDD is carried out using non-invasive procedures and accepted in common clinical practice. To protecting patient privacy throughout the project, the manuscript and databases do not contain names or personal data. Given that it is a non-interventionist trial, retrospective work using information from a database of consecutive cases, the ethics and research committee of Hospital Médica Sur authorizes its implementation, the use of numerical data and the publication of this article without the need of informed consent. We testify that this work is original and is not currently considered for publication elsewhere. Partial data from this work was presented (poster session) at the 24th World Neurology Congress 2019, and a version of the abstract was published in the Journal of the Neurological Sciences 405(2019):314. 10.1016/j.jns.2019.10.1413.

## Results

We studied 21 patients with a first-ever syncope (16 female, 6 male, mean age 43.5 years; max 85, min 20, St. D 17.5) and 21 control subjects (15 female, 7 male, mean age 45.1 years; max 83, min 21, St. D 16.4). In the group with syncope, six subjects smoked, one had moderate alcohol intake and four had controlled high blood pressure. In the control group, seven subjects smoked, four had moderate alcohol intake and four were hypertensive, one of them without treatment. We found no significant demographic differences or risk factors between the control group and the group with syncope, so we infer that both are comparable. The data, according to the tests, can be considered a normal distribution throughout the protocol.

When comparing the results of the means of the data in each stage, we observed differences between subjects with syncope and the non-syncope group. We found significant differences in the following variables through the T and Z tests:

- CBF variables: Mean-vel. in stage 6 (44 vs 52, p = 0.022), and in stage 8 (44 vs 55, p = 0.011). Peak-vel. in stage 8 (80 vs 94 p = 0.011). EDV showed a difference in four stages: stage 2 (28 vs 35, p = 0.026), stage 4 (27 vs 33 p = 0.038), stage 6 (26 vs 34, p = 0.020), stage 8 (25 vs 33, p = 0.025). PI, RI and SD values did not show significant differences.
- Cardiovascular variables, significant differences: HR in stage 6 (82 vs 74, p = 0.013), SBP in five stages: stage 2 (107 vs 120, p = 0.007), stage 4 (106 vs 120, p = 0.005), stage 6 (113 vs 121, p = 0.033), stage 8 (111 vs 132 p = 0.033) and stage 9 (109 vs 123 p = 0.003). DBP in stage 2 (70 vs 76 p = 001) and in stage 8 (75 vs 82, p = 0.003) (Table 1).

**Table 1:**
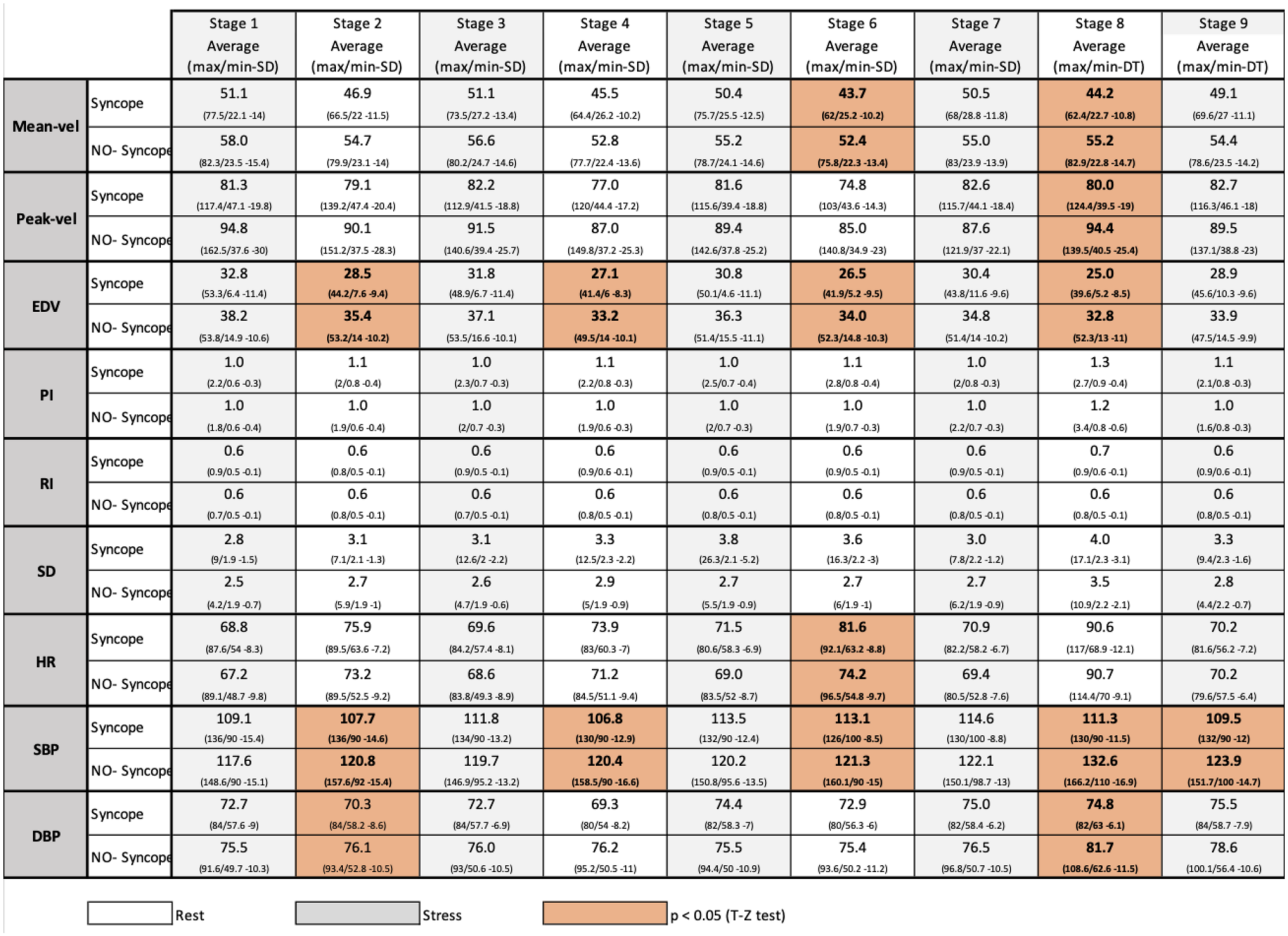
Difference of the mean for each subject in each of the nine stages (Sincope and Non-Sincope groups).

*SBP* systolic blood pressure, *DBP* diastolic blood pressure, *MBP* mean blood pressure, *Peak-vel*. systolic velocity of the MCA, *Mean-vel*. mean velocity of MCA, *EDV* end diastolic velocity of the MCA, *PI* pulsatility index, *RI *resistance index, *SD* systolic/diastolic velocity index.

To describe the differences between stage changes, we use the suffix D in the values. The following was significant according to the T and Z tests: SBP, in changes between positions 1–2, 2–3, 3–4, 4–5, and 7–8. DBP in four changes (1–2, 3–4, 4–5, 7–8). From the 7 to 8 stage, we observe the major difference between the two groups for Mean-vel., Peak-vel., EDV, SBP and DBP variables. At 5–6, the PI and HR variables were different. At 6–7, PI, and at 8–9 were Mean-vel. and Peak-vel. (Table 2).

**Table 2:**
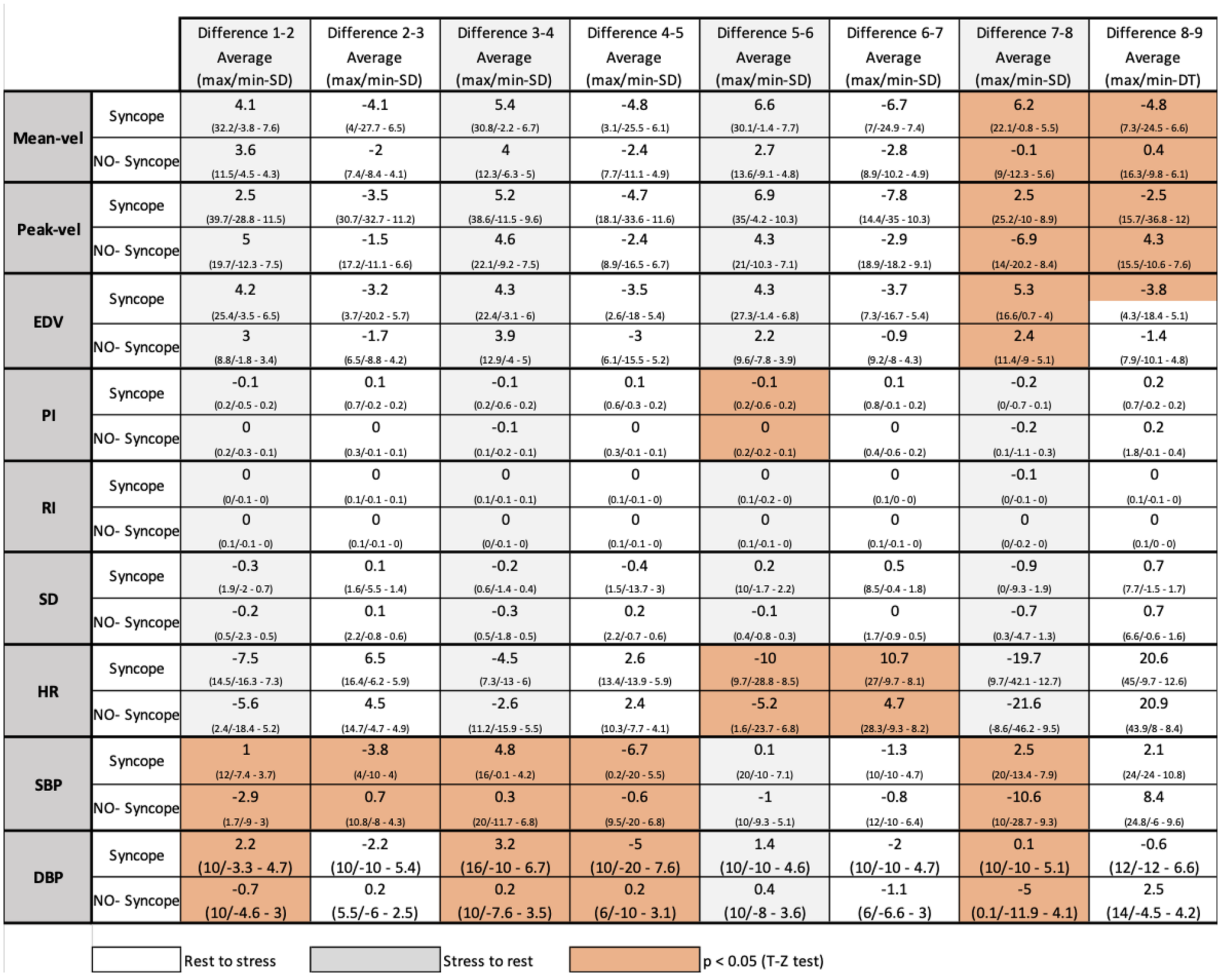
Relationship of the differences of the mean of each variable with consecutive change of stage.

*SBP* systolic blood pressure, *DBP* diastolic blood pressure, *MBP* mean blood pressure, *Peak-vel*. systolic velocity of the MCA, *Mean-vel*. mean velocity of MCA, *EDV* end diastolic velocity of the MCA, *PI* pulsatility index, *RI* resistance index, *SD* systolic/diastolic velocity index.

**Figure 3:**
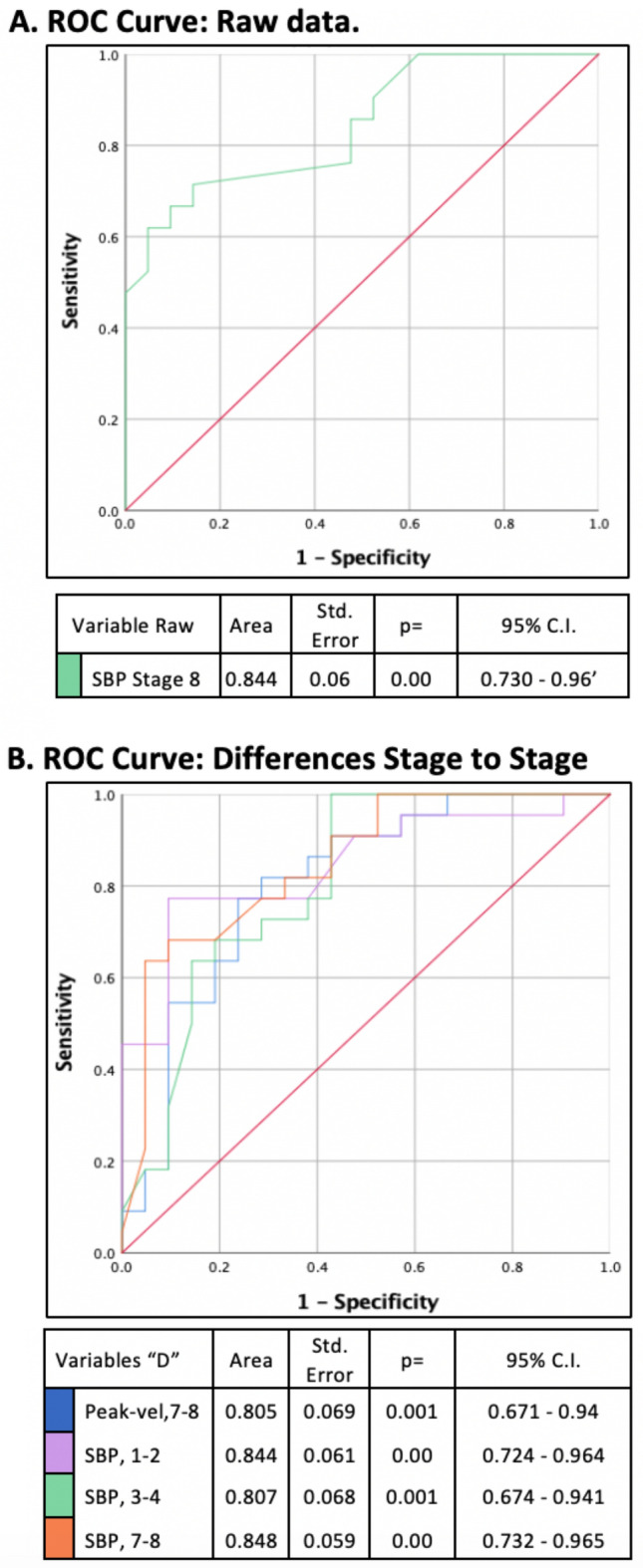
ROC curves of the variables resulting in an area under the curve > 0.70 in a given stage. (**A**) Raw data, (SBP), (**B**) differences stage to stage, (SBP, and Peak-vel) in different stages. *SBP* systolic blood pressure, *Peak-vel*. systolic velocity of the MCA.

Figure 3 shows the ROC curve of those variables with a significative difference in T/Z test, resulting in an area under curve higher than 0.800: Fig. 3A, the raw data of SBP in stage 8, and Fig. 3B, variables of the change between stages, Peak-vel.D in 7–8, and SBP.D in 1–2–3–4–7–8.

These first results confirm the difference between the analyzed groups. As our goal is not to make a predictive model, but to measure the ability to test to find differences, we used the logistic regression test to improve the description of the position changes that contribute to the diagnosis of syncope. Using D values, we obtained the regression coefficients for each combination of variables and for each change of position mentioned above. The sign of the coefficients of the logistic regression describes the changes by the physiological variables between each contiguous stage, while their magnitude indicates the weight of each of them. For the purpose of this work, we only consider the signs. We obtained 160 results and determined the predominant sign of the coefficients of the regression, its statistical significance and the area under the corresponding ROC curve.

**Figure 4:**
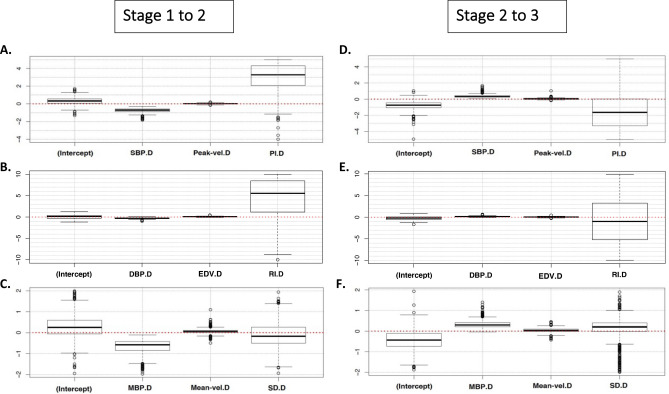
Boxplots of the variation of the coefficients obtained with bootstrap (with 1,000 iterations) with three groups of variables. Stage 1 to 2: From rest to test; in the change from stage 1 (of rest) to stage 2 (of test) Stage 2 to 3. From test to rest; in the change from stage 2 (of test) to 3 (of rest). *SBP* systolic blood pressure, *DBP* diastolic blood pressure, *MBP* mean blood pressure, *Peak-vel*. systolic velocity of the MCA, *Mean-vel*. mean velocity of MCA, *EDV* end diastolic velocity of the MCA, *PI* pulsatility index, *RI* resistance index, *SD* systolic/diastolic velocity index.

As an example, we show the boxplots of the variation of the coefficients obtained with bootstrap (with 1,000 iterations) in the change from stage 1 (of rest) to stage 2 (of test) with three groups of variables (Fig. 4). The sign shows the type of change that a physiological variable should undergo to indicate syncope. (increase or decrease for positive or negative, respectively, is denoted on the horizontal axis by a red line in order to show the different signs) that a physiological variable should undergo to indicate syncope. For example, the coefficients of the variables SBP.D (Fig. 4A), DBP.D (Fig. 4B) and MBP.D (Fig. 4C) always have a negative sign. This indicates that a person with syncope should show a decrease in these variables when the change of position occurs. Figure 4 shows also the coefficients of the physiological variables when going from test (Stage 2) to rest (Stage 3). Here, the variables SBP.D, DBP.D and MBP.D (Figs. 3, 4D,F), in most cases show positive coefficients. The latter would indicate that a person with syncope should show an increase in these variables in the position change we previously mentioned.

**Figure 5:**
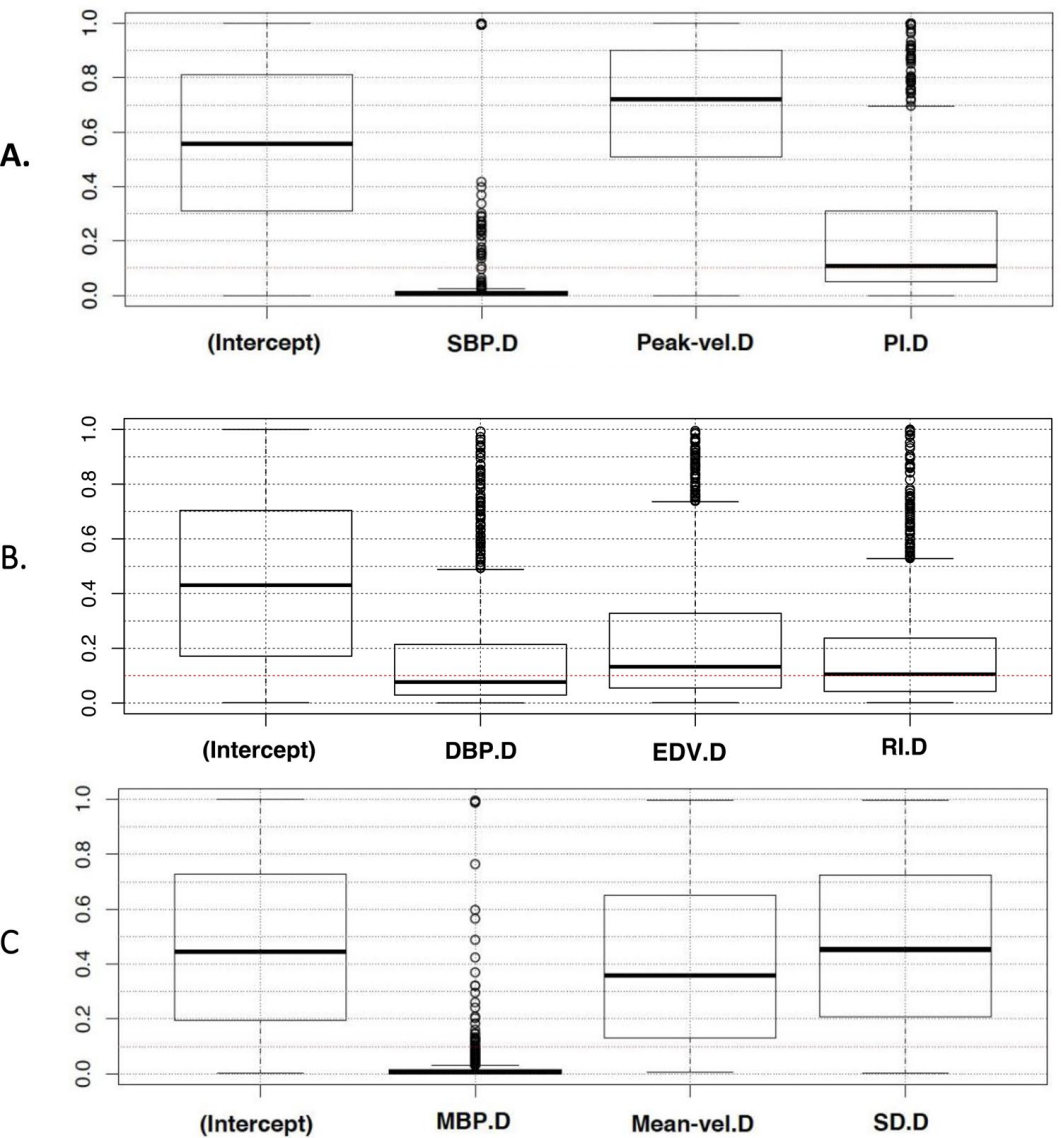
Statistical significance of the coefficients: boxplot showing the significance value of the signs at change 1–2 (bootstrap with 1,000 iterations). (**A**) The variable SBP.D appears highly significant. Peak-vel.D, as a variable with no statistical relevance and PI.D in 50% of cases as a significant variable. (**B**, **C**) Other combinations of the variables with their corresponding coefficients for the change between stages 1–2. *SBP* systolic blood pressure, *DBP* diastolic blood pressure, *MBP* mean blood pressure, *Peak-vel* systolic velocity of the MCA, *Mean-vel* mean velocity of MCA, *EDV* end diastolic velocity of the MCA, *PI* pulsatility index, *RI* resistance index, *SD* systolic/diastolic velocity index.

The variation of the negative sign in change 1–2 to positive in change 2–3 of the variables SBP.D, DBP.D and MBP.D, shows that the values generated by the MMDD are sensitive enough to observe the patient’s syncope when going from rest to test and vice versa. If the patient with syncope is at rest and undergoes a test, the change of their physiological variables must be the opposite when they return to a basal state (normality). To understand the results, we evaluated the statistical significance of the sign from the coefficients of the regression, Fig. 5 shows the p-values of the coefficients obtained by each logistic regression with a bootstrap of 1,000 iterations. The red line indicates where the p-value is 0.1. Figure 5A, shows the boxplots of the significance values of the coefficients in the change at 1–2 using the group of variables (SBP.D, Peak-vel.D, PI.D). The intercept coefficient can be considered equal to zero, whereas the variable SBP.D appears highly significant in most cases. Peak-vel.D is a variable with no statistical relevance. PI.D appears as a significant variable in 50% of the cases. Figure 5B,C show the p-values of the coefficients for the change between stages 1–2 with other different combination variables.

**Figure 6:**
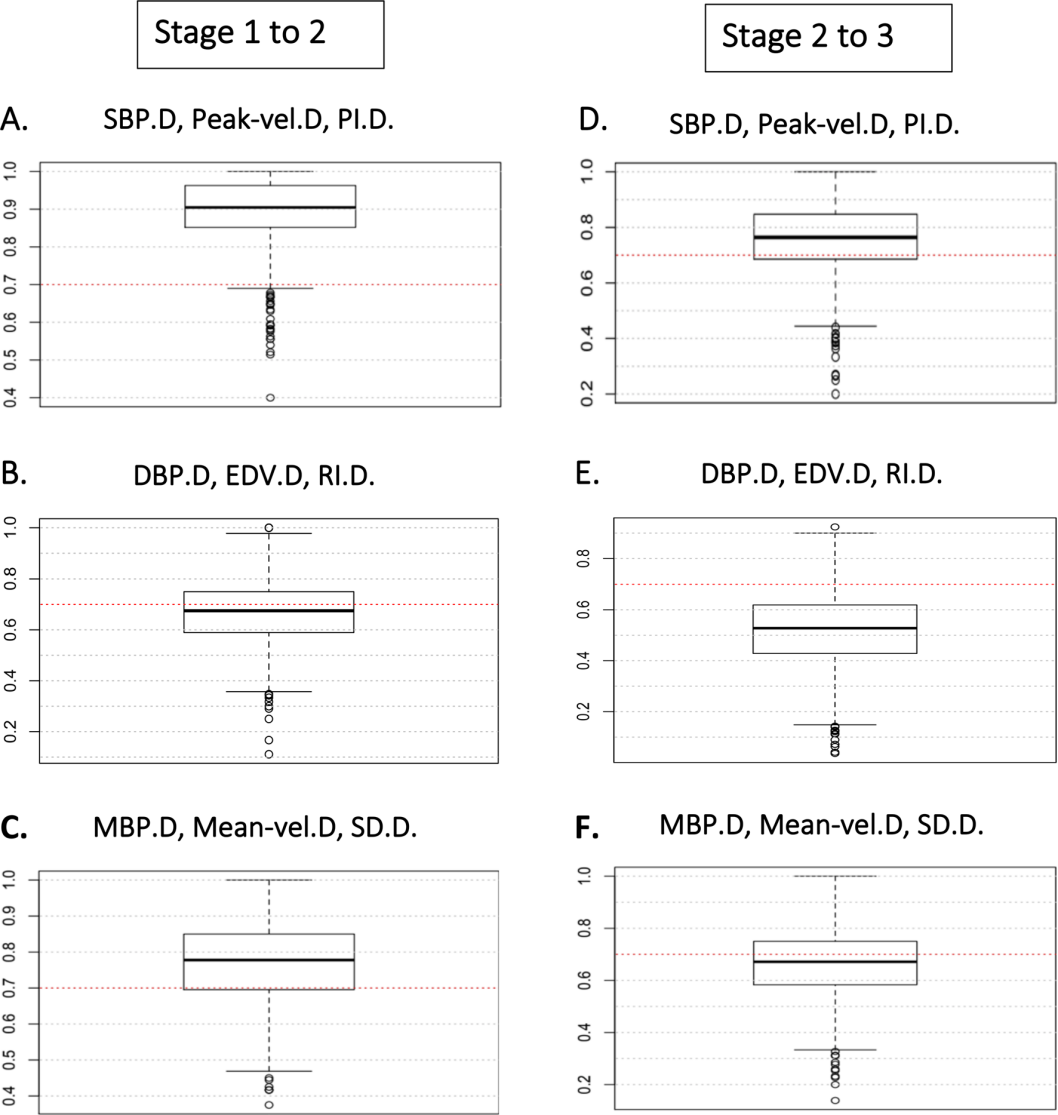
Boxplot showing the values of the area of each generated ROC curve by the bootstrap technique (1,000 iterations). From stage 1 to stage 2 (**A**) shows that (SBP.D, Peak-vel.D, PI.D) are good information to distinguish patients with syncope from stage 1 to stage 2. Meanwhile, in (**B**), (DBP.D, EDB.D, RI.D) is not good, and in (**C**), (MBP.D, Mean-vel.D, SD.D) is regular. From stage 2 to stage 3. (**D**) Shows that (SBP.D, Peak-vel.D, PI.D) is sufficiently good to distinguish patients with syncope. (**E**) shows that (DBP.D, EDV.D, RI. D) is poor to do this, and (**F**) (MBP.D, Mean-vel.D, SD.D) is not good. *SBP* systolic blood pressure, *DBP* diastolic blood pressure, *MBP* mean blood pressure, *Peak-vel.* systolic velocity of the MCA, *Mean-vel* mean velocity of MCA, *EDV* end diastolic velocity of the MCA, *PI* pulsatility index, *RI* resistance index, *SD* systolic/diastolic velocity index.

Figure 6 shows the value of the area under the ROC curve for each generated logistic regression in the bootstrap for each of the 1,000 iterations. When the area under the ROC curve obtained by logistic regression is near to 1, we can assume that the acquired data allows us to discriminate between two conditions, in our case patients with or without syncope, since the ROC curve is a plot of sensitivity vs. fall-out (= 1—specificity). In Fig. 5A,D, the set of variables SBP.D, Peak-vel.D, PI.D allow distinguishing patients with syncope in the transition from stage 1 to stage 2 and from stage 2 to stage 3 since at least 70% obtained values are above 0.7 (red line). Meanwhile, the set DBP.D, EDV.D, RI.D appears not to be sufficient to distinguish syncope in the change from stage 1 to stage 2 (Fig. 5B), and DBP.D, EDV.D, RI.D is bad for stage 2 to stage 3 (Fig. 5E).

While performing bootstrap with 10,000 iterations, we assigned a sign. We gave statistical value when at least 70% of the cases resulted in same-sign coefficients, and 70% of the corresponding p-values were below 0.1. We consider that logistic regression is a good classifier if the area under the ROC curve for 70% of generated ROC curves by the bootstrap procedure is at least 0.7. From our simulation with 10,000 iterations, we created Table 3, where we summarize signs of the coefficients of the logistic regression obtained for each variable while interacting with different groups, their p-values, and areas under the ROC curve.

**Table 3:**
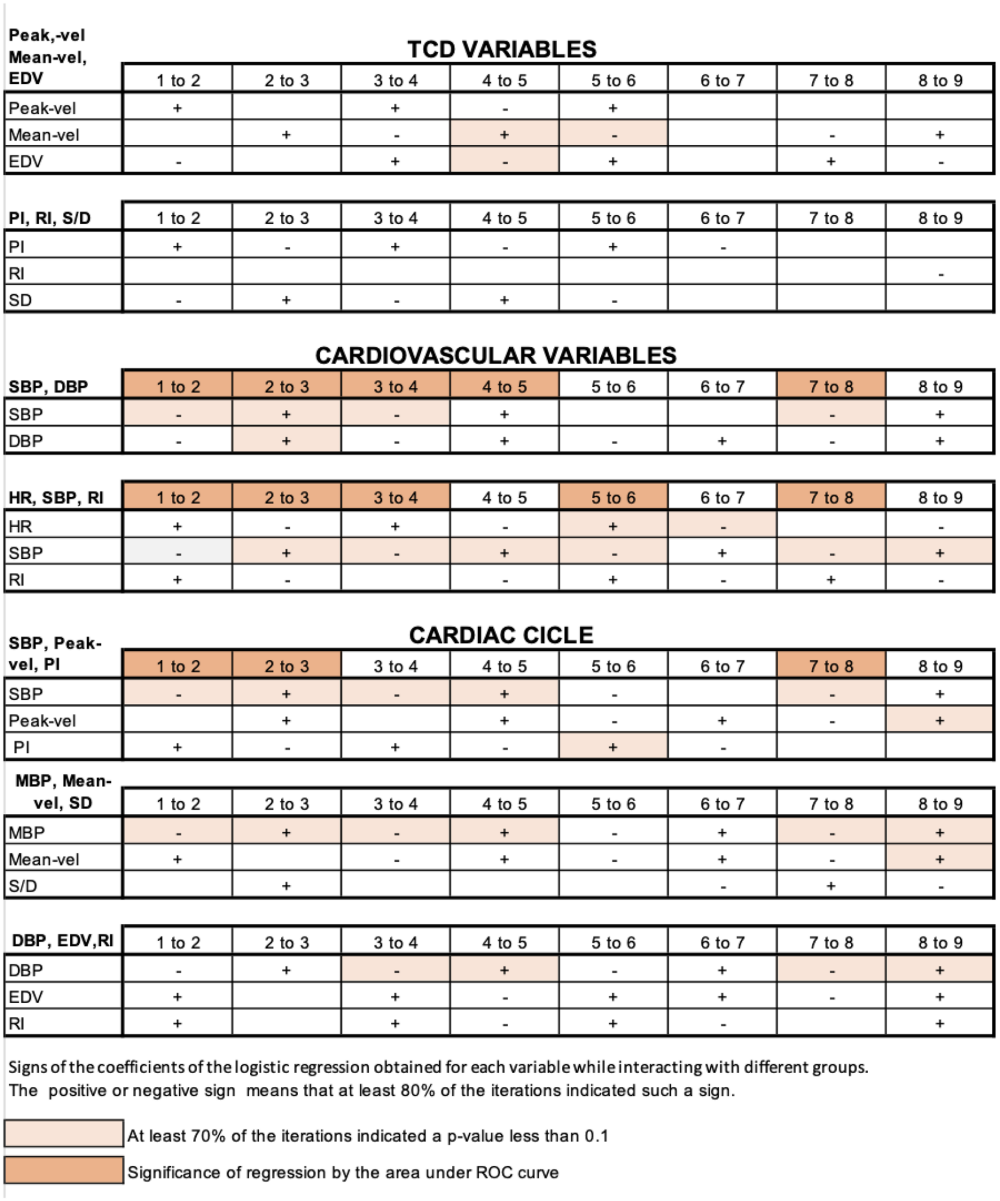
Result of the regression coefficients using Bootstrap with 10 k (implemented in R).

As shown in Table 3, it can be observed the behavior of the sign during MMDD for every change of stage. For example, for the group (Peak-vel.D, Mean-vel.D, EDV.D), the coefficients for Mean-vel.D are changing from positive to negative. In the case of Peak-vel.D, in the same group, they change signs from stages 3 to 6. The signs that achieved statistical significance were the CBF variables in the group (Peak-vel.D, Mean-vel.D, EDV.D) Peak-vel.D and Mean-vel.D have statistical significance at 4–5 and Mean-vel.D for 5–6. For the group (PI.D, RI.D, SD.D), it is possible to determine changes of signs in the different stages, but they did not show a statistical significance. In the group (SBP.D, DBP.D), the signs with statistical significance belong to SBP.D, positive at 2–3 and negative at the changes 1–2, 3–4 and 7–8. For the group (HR.D, SBP.D, RI.D), the signs for HR.D, which reached a statistical significance, are at 5–6 positive and 6–7 negative. In this last group of variables, SBP.D again reached a statistical significance in the changes of 1–2, 2–3, 3–4, 4–5, 5–6 and 7–8, 8–9. Considering variables of the cardiac cycle, in systole (SBP.D, Peak-vel.D, PI.D.), the sign for SBP.D reach statistical significance in 1–2, 2–3, 3–4, 4–5 and 7–8 and for PI.D in 5–6. Considering the group by the diastole (DBP.D, EDV.D, RI.D), only the sign for DBP.D during changes 3–4, 4–5, 7–8 and 8–9, reached statistical significance. Considering the intermediate moment of the cardiac cycle (MBP.D, Mean-vel.D, D/S), the signs for MBP.D achieved statistical significance during 1–2, 2–3, 3–4, 4–5, 7–8, and 8–9 and Mean-vel.D at 8–9.

Respect to the area under the ROC curve, in the groups (SBP.D, DBP.D), we saw a good performance during 1–2, 2–3, 3–4, 4–5 and 7–8. For the group (HR.D, SBP.D, RI.D) in 1–2, 2–3, 3–4, 5–6 and 7–8, and in the group (SBP.D, Peak-vel.D, PI.D) in 1–2, 2–3, 7–8.

## Discussion

Technological advances in the last three decades have been extraordinary. We consider it convenient to search for new options to study patients with ANS disorders. We share this concern with other teams; in a recent paper, using the Calgary scale to stratify the risk of syncope in test subjects, positivity was when compared using the Bruce protocol of the stress test added with 300 mcg of sublingual nitroglycerin to identify the patients with syncope. Although the acquisition of the variables happens during a mobility test, the positive result is given by evoking syncope using a pharmacological challenge^26^. In another work, they use mathematical tools to understand the physiological changes in patients with syncope using the mathematical idea of entropy to analyze the ANS complexity before the moment of a syncope using passive movement in the TT^27^.

In our opinion, the study of syncope and conditions of the ANS requires changes in paradigms. Inspired by the works of Ewing in 1985^10^, we designed the MMDD: a free mobility test that resembles daily life conditions and allows the study of newly available technology, the ANS's behavior in patients with syncope.

In this way, we use pulse-to-pulse blood pressure records with pulse transit time^28^, TCD monitoring and other methods to assess the state of CBF autoregulation^12,29,30^, to obtain in each beat four variables of general circulation (heart rate, systolic, diastolic, and mean arterial pressure) and six variables of CBF (Peak-vel., Mean-vel, EDV, PI, RI, SD).

We also consider the activities we submit our patients to during the study to be close to real-life situations. As we use mathematical modeling to characterize patients with dysautonomia, we do not require to evocate syncope as a criterion of positivity.

In the first statistical analysis, we showed the different means of the variables in each stage of the study between patients with syncope and the control group. Later, using the means between the different D values in the stages, we verified the difference in change suffered by the variables between positions, which is again, different between patients with syncope and the control group. These differences allow us to confirm the hypothesis that, through the MMDD, we obtain data to distinguish ANS's behavior in patients with syncope. We took advantage of the bootstrap technique^31^ to perform up to 10,000 iterations to understand the behavior of the changes of the variables in each change of position through logistic regression. The sign of the coefficients in the logistic regression describes changes suffered by physiological variables induced by changes in position from one stage to another. Considering the groups of the variables mentioned above, we could estimate the probability of suffering a syncope. For this paper, we focus only on analyzing the signs to understand how the changes are in the physiological variables and then give a description of a "standard" patient with syncope. Table 3 summarizes the combination of resulting signs.

Regarding the correlation of our results with previous knowledge: blood pressure has a paradoxical behavior. In patients undergoing TT, at the time before presenting syncope, the systolic blood pressure drops, and the diastolic blood pressure rises^32^. In our study, the variable SBP showed a relevant differentiator between patients with syncope and the control group during almost all the test stages. Moreover, the change between SBP and DBP when moving from one stage to another has a characteristic behavior in patients with syncope at 5–6 and 6–7, wherein some cases one increases and the other decreases, since SBP has no a predominant sign. This observation of different signs shows a "paradoxical" behavior in SBP and DBP interaction with patients without syncope (Table 3).

CBF typically remains stable despite variations in blood pressure thanks to the CBF autoregulation mechanism. Under normal conditions, the sympathetic and parasympathetic autonomic nervous system's innervation has little direct influence on CBF. It depends more on humoral changes in the central nervous system^33,34^. Under normal conditions, changes in blood pressure should not produce significant CBF changes as long as they do not exceed the autoregulation limits.

In neurally mediated syncope and postural orthostatic tachycardia syndrome (POTS), the chronotropic response of the heart is not related to the vasopressor response and may even be paradoxical with the CBF response, suggesting a central control disorder of the latter^35^. In this work, HR's behavior showed differences between patients with syncope and the control group during standing (stage 6) and in stages 5–6 and 6–7. This is corroborated with logistic regression by showing the significance of changing this variable when interacting with others. During the change at 5–6 (from lying to standing), the resulting coefficient for HR has a positive sign (p-value < 0.1) and in 6–7 (from standing to rest) a negative sign (p-value < 0.1), associated with the sign in SBP, that is, at 5–6 h increases and SBP decreases, which indicates syncope.

In cerebral syncope and disorders of the ANS, it is fundamental to consider cerebral vascular reactivity. Different authors have used TCD to study cerebral autoregulation in both animal and human experiments^36^. It is valid for calculating CBF autoregulation's lower limit and sensitive to changes in perfusion pressure in healthy patients^30^. Our focus is to study the relationship between systemic circulation and its influence on CBF. With our method, we found differences in the CBF variables at various moments of the test. For example, EDV patients with syncope are significantly different from those with non-syncope it in all stages of our test: Mean-vel in the standing position and while effort, and Peak-vel at the time of the effort, and differences during 7–8 and 8–9 changes, which are recovery phases. One of the most outstanding findings in this work happens while reviewing the signs of the CBF coefficients: we observed the alternating signs of Peak-vel., Mean-vel, and EDV as the three variables interact. Mean-vel and Peak-vel., for example, have an alternation (opposite). It is possible to see how the patient with syncope modifies these variables in changes of "every day" positions. These changes are a mathematical representation of the behavior of the CBF variables during the test.

## Conclusions

We demonstrate the feasibility of using the MMDD to detect subtle physiological changes during a free mobility trial. As it shows notable differences between patients with and without syncope, we were able to establish the hypothesis that these differences distinguish the behavior of ANS and CBF in patients with syncope.

Through the bootstrap technique, we efficiently performed a multivariate logistic regression study and reviewed the joint behavior of different variables while changing position. The changes of sign in the coefficients of a multivariate logistic regression model from stage to stage describe an individual with syncope. The sign direction is an indicator of what kind of change would be expected in some physiological variables for a "standard" syncope. Thus, meanwhile studying a new patient with MMDD, the certainty that a new patient has syncope increases when the more coincidences exist with our results. This hypothesis is subject to research in our group.

Furthermore, once demonstrated that our analysis permits understanding some particular subtle characteristics of syncope, we are currently testing modifications to the protocol to increase its sensitivity and specificity, and we are working on a clustering model and developing artificial intelligence to propose a new approach for the study of patients with dysautonomia.
